# Risk of Hepatocellular Carcinoma in Workers Exposed to Chemicals

**DOI:** 10.5812/hepatmon.5943

**Published:** 2012-10-11

**Authors:** Mario Uccello, Giulia Malaguarnera, Thea Corriere, Antonio Biondi, Francesco Basile, Mariano Malaguarnera

**Affiliations:** 1 Research Center “The Great Senescence”, University of Catania, Catania, Italy; 2 Department of General Surgery, University of Catania, Catania, Italy

**Keywords:** Carcinoma, Hepatocellular, Occupational Exposure, Vinyl Chloride, Solvents, Pesticides, Polychlorinated Biphenyls, Arsenic

## Abstract

**Context:**

Studies on experimental animals have shown liver is a common target of chemical carcinogens; this might suggest that occupational exposure to chemicals is another risk factor for HCC. However, the relationship between occupation and liver cancer has not been extensively studied, with the exception of the known association between vinyl chloride and angiosarcoma of the liver.

**Evidence Acquisition:**

A MEDLINE and conventional search of the past 50 years of the medical literature was performed to identify relevant articles on incidence and mechanisms of HCC due to occupational exposure to chemicals. Several important edited books and monographs were also identified and reviewed.

**Results:**

While laboratory data clearly indicate that the liver is an important target of chemical carcinogenesis, epidemiological studies provide very limited evidence on occupational risk factors for HCC. Nevertheless, we found some case reports and epidemiological data showing a moderately increased risk of HCC development in people exposed to vinyl chloride, organic solvents, pesticides, polychlorinated biphenyls, and arsenic.

**Conclusions:**

Occupational exposure to chemicals may be another risk factor for HCC development, but the interpretation of currently available findings is limited by the small number of studies, questionable accuracy of the diagnosis of liver cancer, and potential confounding or modifying factors such as chronic hepatitis virus infection and alcohol consumption. Further relevant investigations are required for clarifying the actual contribution of occupational exposure to chemicals in HCC development.

## 1. Context

Toxic environmental exposures, including workplace exposures, are responsible for a substantial number of cancer cases. Exact estimation of this number is not possible because of gaps in the data on exposure, interactions with lifestyle carcinogens, and differences in the exposure patterns in different countries (1). Few studies have assessed occupational exposures. Despite the progressive improvement in measures to reduce workplace carcinogenic exposures in most countries, occupational exposure to carcinogens is still a remarkable health risk worldwide, particularly in developing countries with intense and uncontrolled exposure to common environmental carcinogens (1-3). Hepatocellular carcinoma (HCC) represents a major health problem worldwide. It is the dominant form of primary liver cancer and the third leading cause of cancer-related deaths, accounting for over half a million deaths per year (4-6). Environmental factors play a key role in the development of HCC. Approximately 70%–90% of HCC patients have an established history of chronic liver disease and cirrhosis, whose major risk factors include alcoholic liver disease and chronic infection with hepatitis B virus (HBV) and hepatitis C virus (HCV) (7, 8). Additional risk factors for developing HCC include nonalcoholic steatohepatitis (NASH), intake of aflatoxin-contaminated food, diabetes, obesity, cigarette smoking, certain hereditary conditions such as hemochromatosis, and certain metabolic disorders (9-11).

The relationship between occupation and liver cancer has not been extensively studied, with the exception of the known association between vinyl chloride monomer (VCM) and angiosarcoma of the liver (ASL) (12). However, occupational exposure to toxic chemicals may be associated with a high risk of HCC. This hypothesis seems plausible, especially because the liver is the most common site of tumor origin in experimental animals treated with chemical carcinogens (13). Moreover, some epidemiological studies on cancer incidence or mortality among individuals in occupations that usually involve exposure to chemicals have shown that such individuals have a moderately high risk of primary liver cancer (14-17). According to the latest evaluation reports of the International Agency for Research on Cancer (IARC), only VCM and trichloroethylene (TCE) have been found to have a carcinogenic effect on human liver (Table 1). However, recent data clearly indicate that the liver is a potential target of other occupational carcinogens. In this paper, we have summarized the current knowledge about selected chemicals and their possible role as occupational risk factors of HCC.

**Table 1:** Brief Summary of the Latest International Agency for Research on Cancer (IARC) Evaluation Reports on the Carcinogenicity of Vinyl Chloride, Arsenic, Trichloroethylene, Perchloroethylene, Polychlorinated Biphenyls, and 1,1,1-Trichloro-2,2-Bis(p-chlorophenyl)Ethane (DDT)

	Year of Publication	Group Assigned by IARC	Summary of IARC Evaluation on Overall and Liver Carcinogenicity a
**Vinyl chloride**	2008	Group 1 (carcinogenic to humans)	There is sufficient evidence for the carcinogenicity of vinyl chloride because it has been found to induce angiosarcomas of the liver and hepatocellular carcinomas in both humans and experimental animals (21).
**Arsenic**	2004	Group 1 (carcinogenic to humans)	There is sufficient evidence in humans that arsenic in drinking water causes cancers of the urinary bladder, lung, and skin; however, the interpretation of the findings on liver cancer mortality has several methodological limitations. Moreover, the studies on inorganic arsenic provide limited evidence for carcinogenicity in experimental animals (102).
**Trichloroethyl**ene****	1995	Group 2A (probably carcinogenic to humans)	Evidence from several epidemiological studies suggests that trichloroethylene may enhance the risks for non-Hodgkin’s lymphoma and cancers of the liver and biliary tract. Trichloroethylene can also induce liver tumors in rodents through peroxisome proliferation and other mechanisms (40).
**Perchloroethy**lene****	1995	Group 2A (probably carcinogenic to humans)	There is consistent evidence for positive associations between exposure to tetrachloroethylene and the risks for non-Hodgkin’s lymphoma and esophageal and cervical cancer. Although tetrachloroethylene is known to induce hepatocellular carcinomas in experimental animals, evidence of liver tumor induction in humans is inconsistent (40).
**Polychlorinated biphenyls**	1987	Group 2A (probably carcinogenic to humans)	Evidence for carcinogenicity in humans is limited. An increased risk from hepatobiliary cancer has been found in different studies, which were biased. On the other hand, evidence for carcinogenicity in animals is sufficient. Administration of certain polychlorinated biphenyls produced benign and malignant liver neoplasms in mice and rats (25).
**DDT**	1991	Group 2B (possibly carcinogenic to humans)	There is inadequate evidence for the carcinogenicity of DDT in humans. Evidence for the carcinogenicity of DDT in experimental animals is sufficient, particularly for liver tumors (121).

^a^After the publication of the last IARC monographs, there has been more evidence regarding the carcinogenic effect of these chemicals on the liver.

## 2. Evidence Acquisition

A MEDLINE and conventional search of the past 50 years of the medical literature was performed. In order to identify relevant articles on incidence and mechanisms of HCC due to occupational exposure to chemicals, we first performed a MEDLINE search of articles from 1966 onward. Secondly, we searched the reference lists of the articles initially retrieved for additional studies. This method of cross-checking was continued until no further publications were found. In case of multiple publications on the same study population, we used the most recent publication. Several important edited books and monographs were identified through “Web of Science” searching, bibliography review, and expert consultation. Particular emphasis was placed on the “IARC Monographs on the Evaluation of Carcinogenic Risks to Humans”, performed by international working groups of independent scientists and often used as the basis for regulatory classification. Nevertheless, we took care of including the evidence emerged after the publication of the latest IARC monographs. With regards to representative animal studies, epidemiological investigations, case reports and other relevant data, only reports that have been published or accepted for publication in the openly available scientific literature were reviewed. Material provision was conducted independently by each author, and any discrepancies among reviewers were resolved by consensus. Eligible material was selected for evaluation. Language other than English was not an exclusion criterion. Cases of liver injury or HCC from causes not closely related to occupational or environmental exposures to chemicals were not included.

### 2.1. VCM and Polyvinyl Chloride

**Figure 1:**
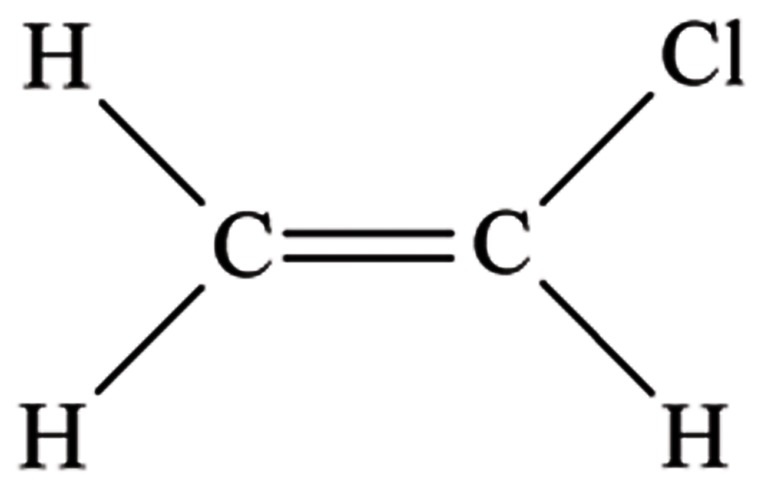
Chemical Structure of Vinyl Chloride Monomer

VCM is a chlorinated organic compound (Figure 1); at room temperature and pressure, VCM is a typically sweet smelling colorless gas insoluble in water. It is mainly used in the production of the polymer polyvinyl chloride (PVC) (18). VCM is rapidly absorbed after respiratory exposure and is primarily metabolized by the liver (19). The metabolic pathway for the elimination of VCM generates intermediate metabolites that can also form mutagenic DNA adducts (20). Moreover, the production of VCM and PVC involves the use of various chemicals, some of which are carcinogenic (i.e. ethylene dichloride) (21). VCM is extensively used as a refrigerant in the plastic-manufacturing industry and as an intermediate in organic synthesis (22). Before the implementation of environmental controls, VCM exposure levels were high, measuring up to 13000 ppm (23). Nowadays, in developed countries where strict controls have been instituted, the levels of occupational exposure to VCM are usually less than 1 ppm (24). Evidence from a wide range of experimental and epidemiological studies has shown the carcinogenicity of VCM in animal models and humans (20). Therefore, VCM has been classified as a group I carcinogen by the IARC (18, 25). There is considerable evidence about the association between occupational exposure to VCM and liver cancer (15), as well as tumors of the brain (26), lungs (27), and hemolymphopoietic system (28). VCM is known to cause ASL, but it can also exert other toxic effects on the liver, including cirrhosis and HCC (29). VCM exerts a directly toxic effect on the liver (19), and it may also cooperate with other risk factors to cause liver injury (29, 30). VCM causes damage mainly at the sinusoidal level, but hepatocytes may also be involved (12, 31). Maroni et al. (32) have reported that the findings of liver ultrasonography in VCM-exposed workers showed abnormalities such as hepatomegaly, steatosis, and periportal fibrosis, with a frequency of occurrence higher than the expected range in the general population. Furthermore, a high prevalence of Kras2 mutations (33) and a characteristic p53 mutation pattern (34) have been observed in VCM-exposed workers with HCC. In 2003, Boffetta et al. (15) conducted a meta-analysis on studies of occupational exposure to VCM and its relationship with cancer mortality. The meta-analysis included most of the previously studied workers populations in the United States, Europe, and Asia. Apart from the known risk of ASL, workers exposed to VCM were found to have a high risk of HCC. Many investigations have analyzed a cohort of Italian blue-collar workers in a VCM petrochemical plant in Porto Marghera (Venice, Italy) (35-37). Using a group of workers with low (or null) exposure to VCM as an internal reference, Gennaro et al. (35) reanalyzed the cohort of blue-collar workers during the follow-up period from 1972 to 1999. Mortality from liver cancer, including ASL and HCC, was high in autoclave workers. In conclusion, the epidemiological and experimental studies have reported sufficient data supporting an association between inhalation exposure to VCM and HCC (21) (Table 1).

### 2.2. Organic Solvents

Occupational exposure to various types of organic solvents, such as chlorinated, aromatic, aliphatic, or alicyclic hydrocarbons, is common in many industrial sectors. High risk of mortality from cancer of the liver and biliary passage was found in a meta-analysis of cohort studies among workers generally exposed to organic solvents (38). However, few studies have been conducted on the association between the exposure to individual solvents and the risk of developing liver cancer, with the exception of trichloroethylene (TCE) and perchloroethylene (PCE). TCE, a nonflammable colorless liquid, is an important environmental and industrial pollutant. During the past decades, it has been widely used as an industrial solvent and degreasing agent (39). Because TCE is a common contaminant of groundwater and air, its potential in adversely affecting human health is being studied and debated; however, its worldwide industrial use has decreased over time. Occupations involving TCE exposure include aircraft/aerospace maintenance or manufacture and other industries, including metal degreasing, shoe manufacturing, painting, dry cleaning, electronics, printing, and chemical industries (40, 41). Although TCE exposure has been associated with a wide array of adverse health effects, its toxicity mainly affects the kidney and liver (42). The possible carcinogenicity of TCE remains a controversy. Experimental studies have shown high risks of kidney, liver, lung, and hematopoietic neoplasms among TCE-exposed animals from different species (43). However, discordance in the results of the studies and species-specific differences in TCE metabolism have limited the extrapolation of these findings to human carcinogenesis (44, 45). TCE exposure, for instance, causes HCC in mice but not in rats (46). The precise modes of action (MOAs) by which TCE exposure causes liver tumorigenesis are presently unknown. However, such mechanisms might include: peroxisome proliferation induction; oxidative stress; alterations in cell replication, selection, or apoptosis as a result of cell-signaling; and DNA mutagenic effects (47). Laboratory studies indicate that TCE is not the only chemical responsible for the occurrence of tumors in TCE-treated mice; its 2 metabolites, dichloroacetic acid and trichloroacetic acid, also cause such tumors, probably via different MOAs (49). A slightly increased risk of developing liver cancer has been found among workers exposed to TCE, but inconsistencies in findings across the study populations and some methodological limitations make the currently available epidemiologic data insufficient in supporting a causal relationship between occupational exposure to TCE and HCC in humans (16, 39, 41, 43). (Table 1)

PCE, also known as tetrachloroethylene, is an important chemical primarily used in the dry-cleaning and textile industries and for metal degreasing (48). Many studies have provided clear evidence that both acute and chronic exposure to PCE can cause gastrointestinal disturbances and numerous adverse effects on the central nervous system, kidney, liver, and upper respiratory tract (49-53). In the dry-cleaning industry, the highest exposure occurs during the operation of the machinery, primarily via inhalation and dermal contact (54). An augmented risk of liver cancer has been reported among workers and experimental animals potentially exposed to PCE (55-57); however, the presently available data on this association is inconclusive. IARC has defined PCE as probably carcinogenic to humans (group 2A) working in the dry-cleaning industry (40) (Table 1). The findings of a hospital-based case–control study conducted in northern Italy during 1997–1999 suggest that occupational risk factors for exposure to xylene and toluene play a role in the development of liver cancer (58).

### 2.3. 1,1,1-Trichloro-2,2-bis(p-chlorophenyl)Ethane (DDT) and Other Pesticides

**Figure 2:**
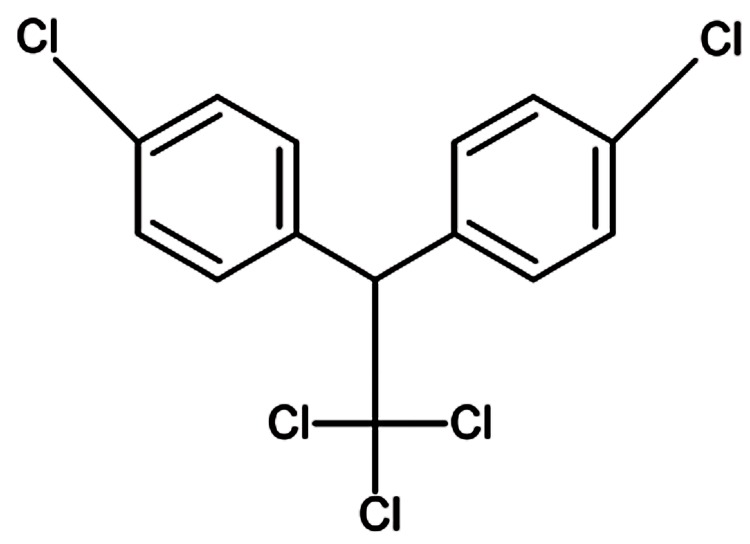
Chemical Structure of 1,1,1-Trichloro-2,2-bis(p-chlorophenyl) ethane (DDT)

Many epidemiological studies have suggested that pesticides play a role in HCC development among people employed in the agriculture industry and who routinely use chemicals to control insects, weeds, rodents, and fungal infections of crops and livestock (14, 17, 59, 60). Moreover, various pesticides such as dieldrin (61), lindane (24), chlordane (62), heptachlor (62), and pyrethrins (63) are established carcinogens in animal models, because the administration of high doses of these pesticides clearly induces liver tumors in some species. However, few investigations have aimed to verify the association between individual pesticides and the risk of liver cancer, except for DDT (Figure 2). DDT is a potent insecticide that was largely used for agricultural and public health purposes from the 1940s to the 1970s; however, concerns about its toxic effects and environmental persistence led many developed countries to ban its use (64). However, DDT is still widely used in Asia and Africa for disease-vector and termite control and as an agricultural insecticide (65). Furthermore, the recommendations and guidelines of the World Health Organization allow the use of DDT to control disease vectors, until suitable alternatives are available (66). The general population is exposed to DDT, primarily through food ingestion, whereas occupational exposures are mainly by inhalation and dermal contact (67, 68). The compound is preferentially stored in the adipose tissue, but the uptake of DDT by fat is slow. Thus, DDT is mostly distributed to other tissues following exposure to a single large dose and to adipose tissue following many small doses. Despite the affinity of DDT for adipose tissue, most of the DDT-related compounds in blood are bound to proteins (69). DDT and its metabolite, 1,1-dichloro-2,2-bis(p-chlorophenyl)ethylene (DDE) have been shown to produce numerous adverse health effects in humans, including neurological, carcinogenic, reproductive, and developmental disorders (67). On the basis of the evidence obtained from animal studies, DDT has been classified as a possible carcinogen (group 2B) by IARC (70) (Table 1) and as a reasonably anticipated human carcinogen by the US National Toxicology Program (71). Similar to many other chlorinated compounds, DDT produces liver cancer in laboratory animals (61, 69). Humans exposed to DDT at work have been reported to have a high risk of liver cancer (59, 72). Furthermore, an ecological study (73) has found a statistically significant correlation between the amount of DDE in adipose tissue and liver cancer mortality rates in white men but not in white women or black men. In a nested case–control study (74), the risk of liver cancer was higher in Chinese men with high blood levels of DDT than those with low levels of DDT. Some epidemiological studies on individuals exposed to DDT have suggested that DDT is hepatocarcinogenic in humans; however, the interpretation of the currently available data is difficult because of the lack of association found among other studies and methodological limitations (67, 74-77).

### 2.4. Polychlorinated Biphenyls

**Figure 3:**
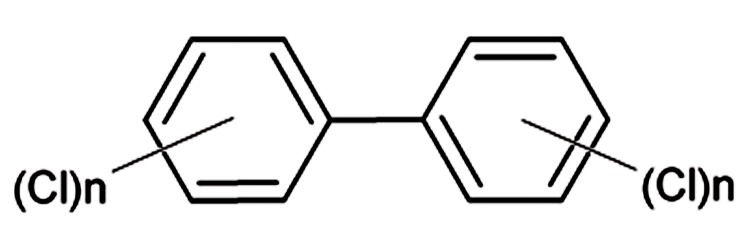
Chemical Structure of Polychlorinated Biphenyls

Polychlorinated biphenyls (PCBs) are synthetic chlorinated aromatic hydrocarbons, which are considered persistent organic pollutants. Generally, all PCBs have a biphenyl molecule (2 attached benzene rings) with a minimum of 1 (but no more than 10) chlorine atoms (Figure 3). The chemical formula for PCBs is C_12_H_10-x_Cl_x_, and 209 different PCB congeners are theoretically possible, depending upon the placement of chlorine atoms; however, only about 130 have been found in commercial PCB mixtures (78). PCBs were widely produced commercially in the USA from 1929 to 1977 and used as electrical insulating, heat exchange, and lubricating fluids because of their high stability, dielectric properties, resistance to oxidation, and incombustibility. PCBs were also blended with other chemicals, such as plasticizers and fire retardants, and used in a variety of products including caulks, adhesives, plastics, and carbonless copy paper. The production of PCBs was highest in the 1970s and had steadily declined thereafter because many countries worldwide have banned the use of PCBs or limited their production (79, 80). Nevertheless, these compounds are still in use and are human health hazards and pose a risk of potential occupational exposure, particularly to people engaged in the maintenance of electrical equipment (78, 81). Most PCBs do not exert particularly acute toxicity, but their persistence, lipophilicity, and propensity to storage in the adipose tissue raise concern over their long-term effects. In animals and humans, chronic exposure to PCBs produces a variety of effects, including reduced body weight, chloracne, edema, liver hypertrophy, porphyria, estrogenic activity, immunosuppression, and neurotoxicity (79, 82). IARC has classified PCBs as a probable human carcinogens (group 2A) on the basis of the evidence of carcinogenicity observed in animals; however, limited evidence has been obtained from human studies (25) (Table 1). Many animal studies conducted by the US National Toxicology Program and others have shown that PCBs play a role in the development of liver disease (83-87). These studies have shown that the liver is the principal target organ for these compounds. Occurrence of benign (toxic hepatopathy, including steatosis) and malignant (HCC and cholangiocarcinoma) liver lesions depends on the dose of the carcinogen. Although laboratory data clearly indicate that PCBs promote activity in the liver and induce preneoplastic lesions and HCCs in animals in a dose- and time-dependent manner, their precise mechanism of action is not known. Many MOAs have been proposed, including direct effects on signal transduction pathways, induction of oxidative stress, effects on vitamin A metabolism, and effects on intercellular communication (88). PCBs are also associated with elevated levels of human alanine transaminase and increased hepatic gene expressions of genes involved in apoptosis, inflammation, and oxidative stress (86). In contrast to the findings of animal studies, data on PCBs in liver diseases in humans are lacking. During a 24-year follow-up after the “Yucheng” incident in Taiwan, where cooking oil was contaminated by PCBs, people of Yechung exposed to high amounts of PCBs showed high mortality from chronic liver disease and cirrhosis (89, 90). While other epidemiological studies have reported an increased mortality from HCC and other cancers among subjects heavily exposed to PCBs (91), Yucheng individuals mortality rates from all malignant neoplasms and liver cancer was not different from those of the general population (90). Moreover, epidemiological evidence shows a link between occupational exposure to PCBs and the risk of HCC. Several studies have investigated the health effects of PCBs among cohorts of electrical capacitor and transformer manufacturing workers and have found a relationship between cumulative exposure to PCBs and liver cancer mortality; however, the hepatocarcinogenesis of PCBs in humans is still controversial (92-95).

### 2.5. Arsenic

Arsenic (As), a natural metalloid element, is considered one of the most significant health hazards in the environment. Groundwater contamination with inorganic As is the primary route of exposure. Exposure to As also results from consumption of contaminated food, contact with contaminated soil, or inhalation of As at work (96-99). Furthermore, arsenicals have been increasingly used in the medical treatment of some types of neoplasms (100). Occupational exposure to As can occur in a number of work settings such as As pesticide manufacturing and use, As refining, glassware factories, semiconductor fabrication, extractive mining, and copper smelting (99). In occupationally exposed subjects, urine and blood samples can be used to estimate the individual doses of ongoing exposure, whereas hair and nail samples can be used to determine the average exposure over a long duration of time (98). Data from epidemiological studies suggest that As exposure is associated with a large number of adverse health effects in various organs and tissue systems, including the cardiovascular, dermal, endocrine, neurological, reproductive, respiratory, hepatic, hematological, renal, and gastrointestinal systems (101). Moreover, after reviewing experimental and epidemiological data, IARC has classified As as a group 1 carcinogen (Table 1). Chronic exposure to As is associated with cancers of the skin, lung, urinary bladder, and possibly liver, kidney, and prostate in humans (102, 103). When considering the carcinogenicity of As, it is important to distinguish between exposure to inorganic and organic forms of As, because only exposure to inorganic As compounds has been associated with cancer (104). Organic As is most abundant in food, particularly seafood, whereas inorganic As compounds are found mainly in drinking water (105). Chronic As exposure causes preneoplastic lesions and liver dysfunctions such as abnormal liver function, hepatomegaly, hepatoportal sclerosis, liver fibrosis, cirrhosis, and ASL (106). While drafting the last IARC monograph, the evidence for As as a liver carcinogen in experimental models was considered limited (102). However, recent laboratory data have shown that the liver is a major target for inorganic As carcinogenesis in rodents and cell model systems (107-111). The exact MOAs of As carcinogenicity, including hepatocarcinogenesis, are not clearly defined, and various potential mechanisms have been postulated (107). These mechanisms include genetic and epigenetic mechanisms such as oxidative DNA damage (112), acquired tolerance to apoptosis, (113) enhanced cell proliferation (114), abnormal DNA methylation (108), genomic instability (115), and aberrant estrogen signaling (116). An increased risk of HCC and ASL in association with environmental exposures to As has also been extensively reported in epidemiological studies and case reports. Many of these investigations have been performed among Asiatic cohorts of people consuming water with high levels of As contamination (117-120).

## 3. Results

Accumulating laboratory findings have clearly indicated that the liver is an important target of carcinogenesis caused by exposure to various chemicals. Compared to experimental studies, epidemiological studies provide very limited evidence on occupational risk factors for HCC. Nevertheless, according to the IARC working group, such association is particularly clear with regard to VCM. Indeed, the incidence of HCC, ASL and liver cirrhosis significantly increases with cumulative occupational exposure to VCM. Moreover, we found some case reports and epidemiological data showing a moderately increased risk of HCC development in people exposed to, organic solvents, pesticides, PCBs, and As. However, the interpretation of these findings is limited by the small number of studies available, questionable accuracy of the diagnosis of liver cancer due to the lack of histological or other definitive clinical data in discriminating HCC from ASL and/or secondary neoplasms, and potential confounding or modifying factors such as chronic hepatitis virus infection and alcohol consumption. Further relevant investigations are required for clarifying the actual contribution of occupational exposure to specific chemicals in HCC development.

## 4. Conclusions

Regardless HCC, prolonged exposure to toxic substances may result in the setting of liver disease. Therefore, it would be reasonable that workers heavily exposed to hepatotoxic chemicals undergo health surveillance, especially in the presence of other established risk factors. The monitoring of liver function should be particularly aimed in detecting any alteration in transaminases, gamma-glutamyltransferase, alkaline phosphatase, and bilirubin levels. In case of marked alterations of these indices, even in the absence of cirrhosis, alpha-fetoprotein levels measurement and hepatic ultrasound examination should be implemented. Such surveillance may well facilitate the early detection of toxic liver diseases (including HCC) in workers exposed to chemcals.
